# Influence of Social Determinants of Health on the Quality of Life of Older Adults in Europe: A Sex Analysis

**DOI:** 10.21203/rs.3.rs-3401316/v1

**Published:** 2023-10-10

**Authors:** Rafael Llorens-Ortega, Carmen Bertran-Noguer, Dolors Juvinyà-Canals, Josep Garre-Olmo, Cristina Bosch-Farré

**Affiliations:** Autonomous University of Barcelona; University of Girona; University of Girona; University of Girona; University of Girona

**Keywords:** population aging, quality of life, social determinants of health, gender perspective, gender equity, longitudinal study

## Abstract

**Introduction:**

The global aging population poses challenges for society such as health inequalities among older persons and between genders.

**Objectives:**

To determine how Social Determinants of Health (SDH) influence the quality of life (QoL) of individuals over 50 years old in various European countries, taking a gender perspective in a longitudinal study.

**Materials and methods:**

Sample of 11,493 individuals from 13 European countries from Waves 5 (2013), 6 (2015), and 7 (2017) of the SHARE study. Instruments: CASP-12 (QoL), EURO-D (depression), SDH: gender, age, educational level, socioeconomic status, ethnicity, place of residence, and European region. Sociodemographic and clinical variables. Statistical analysis: Bivariate and multivariate mixed linear models.

**Results:**

The bivariate analysis showed higher economic hardship and lower education in women compared to men. The CASP-12 score was higher in men than in women. In the multivariate analysis, the variables associated with lower QoL scores among men and women from Wave 5 to Wave 7 were: (β:−0.196, 95% CI: −0.345; −0.047) vs (β:0.038, 95% CI: −0.122; 0.197); economic hardship; and the European region between South and North (β: 2.709, 95% CI: 2.403; 3.015) vs men (β: 2.224, 95% CI: 1.896; 2.551).

**Conclusions:**

The main SDH associated with poorer QoL were female gender, advanced age, economic hardship, educational level, and geographic location within Europe. Depression in women and in Southern Europe were associated with a decrease in QoL scores.

## Plain English Summary

Population aging is a global phenomenon due to the decrease in birth rates and the increase in life expectancy. This demographic shift poses a challenge to society, requiring adaptation of policies, economies, and healthcare systems to safeguard the well-being of older individuals. Health inequalities, influenced by social determinants such as gender, education, socioeconomic status, and place of residence, impact this population group.

Various studies have demonstrated that social determinants of health affect the quality of life of older individuals, especially women. Women generally report lower quality of life indicators, lower educational levels, and poorer health compared to men. This study aims to examine how social determinants of health (SDH) can influence the quality of life (QoL) of the population aged 50 or older in different European countries, from a gender perspective, through a longitudinal approach.

The main SDH associated with poorer QoL were female gender, advanced age, economic hardship, educational level, and geographic location within Europe. Depression in women and in Southern Europe were associated with a decrease in QoL scores.

## Introduction

Population aging is a global phenomenon that is transforming the demographic structure due to declining birth rates and increasing life expectancy [1, 2]. According to the World Health Organization (WHO), it is expected that by 2050, 22% of the global population will be over 60 years old, compared to 12% in 2015 [3]. This shift represents a considerable challenge for society [4]. To protect the well-being of older people and promote healthy and fulfilling aging, countries must adapt their policies, economies, social structures, and health care systems [5, 6]. It is important to address health inequalities that affect this population group, which may be influenced by social determinants of health (SDHs), such as sex, age, educational level, socioeconomic status, ethnicity, race, and place of residence, both internationally and within each country [7, 8].

In 2008, the Commission on Social Determinants of Health defined SDHs as a set of personal, social, economic, and environmental factors that influence the health status of individuals and populations, becoming the most widely used model in SDH research [9, 10]. These determinants have an impact on opportunities for good health and highlight the existence of sex-based health inequalities [11] based on power, prestige, and access to resources [12]. Recent studies have demonstrated a direct relationship between SDHs and quality of life (QoL) among individuals with fewer economic resources [13], those who are older, and women [14]. These determinants influence the QoL of older people, which is a multidimensional construct essential for social well-being and satisfaction of basic needs. QoL encompasses both objective and subjective aspects of the individual, and the WHO defines it as “an individual’s perception of their position in life in the context of the culture and value systems in which they live and in relation to their goals, expectations, standards, and concerns,” which is widely accepted by most authors [15].

Several studies have been conducted on how SDHs influence the QoL of older people in Europe and have shown that objective aspects such as multimorbidity [16], chronic diseases [17], limitations in activities of daily living [18], the presence of depression [18], and lack of material resources [18] have a significant negative impact on their QoL. It is necessary to consider the inequality generated by structural determinants such as socioeconomic status and sex in relation to the QoL of older people, especially women, who generally report worse QoL indicators, lower education levels, and poorer health than men [19]. Recently, the influence of family trajectories on perceived QoL in old age has been studied in Spain using the Spanish cohort of the Survey of Health, Ageing, and Retirement in Europe (SHARE) in waves 3 (2009) and 7 (2017), and it was found that subjective aspects such as social relationships and life as a couple affect the QoL of men and women differently, whereby men with stable partners and offspring perceived a better QoL compared to women in similar conditions [20].

Although previous studies on SDHs and their influence on QoL have been conducted, no specific research has been conducted among people aged 50 years and older from different European countries from a sex perspective to analyze how SDHs can affect their QoL. Therefore, the aim of this study was to examine how SDHs can influence the QoL of the population aged 50 years and older in different European countries from a sex perspective over a four-year follow-up period.

## Materials and methods

This was a population-based, analytical, and prospective cohort study that used data collected in the fifth, sixth, and seventh waves of the SHARE study conducted in 2013, 2015, and 2017, respectively [21]. The information was obtained through computer-assisted personal interviewing (CAPI) with an approximate duration of 90 minutes conducted in the home of each participant and uniformly for all participants [22].

### Participants

In the fifth wave of the SHARE study conducted in 2013, a total of 59,421 individuals from 13 selected European countries were surveyed, including Germany, Austria, Belgium, Denmark, Slovenia, Spain, Estonia, France, Italy, Luxembourg, Sweden, Switzerland, and the Czech Republic. The selection criteria for this study required participants to be 50 years or older, reside regularly in one of the 13 European countries analyzed in the fifth wave, agree to participate in this study, and have participated in the three consecutive waves under study. Of the respondents, 11,493 met these inclusion criteria. The remaining participants did not participate in the consecutive waves or in any of them due to dropout or death [23].

The countries were grouped according to the four regional clusters defined in a 2013 report by the European Commission, which correspond to different models of social welfare: Northern Europe, with a social democratic regime (Denmark and Sweden, n = 2,747); Continental Europe, with a corporatist regime (Austria, Germany, Belgium, France, Luxembourg, and Switzerland, n = 4,443); Southern Europe, with a southern European regime (Spain and Italy, n = 2,770); and Eastern Europe, with a postsocialist regime (Slovenia, Estonia, and the Czech Republic, n = 1,533) [24].

### Study variables

#### Dependent variable

This study assessed participants’ quality of life (QoL) using the control, autonomy, satisfaction, and self-realization (CASP-12) scale [25], a specific and validated tool consisting of four subscales with three items each: control, autonomy, satisfaction, and self-realization. Each item is rated on a Likert scale from 1 (never) to 4 (often). The total score ranges between 12 and 48 points, with a higher score indicating better QoL. Scores below 35 indicate low QoL, 35 to 37 indicate moderate QoL, 38 to 39 indicate high QoL, and 39 to 48 indicate very high QoL. This scale has a Cronbach’s alpha coefficient of 0.84 [26].

#### Independent variables

Data on SDHs were collected through questions related to sex (male, female), age range (50–64 years, 65–74 years, 75–84 years, and over 85 years), educational level according to the International Standard Classification of Education (ISCED) (high, medium, low) [27, 28], socioeconomic level (economic difficulties making ends meet and receiving external financial assistance), ethnicity or race based on the person’s origin (native or immigrant), place of residence (urban, rural, or suburbs of a large city), and European region (north, continental, south, and east).

#### Covariates

Sociodemographic variables included data on marital status (married, divorced, single, widowed), employment status (retired, employed, unemployed, disabled, homemaking), family composition (living alone, living with a partner, living with 3 or more people), number of children (no children, 1 to 2, 3 or more), and number of grandchildren (no grandchildren, 1 to 4, 5 or more).

Clinical variables, Self-perceived health (excellent/very good, good, fair, poor), number of chronic diseases (none, 1–2, 3 or more), difficulties in performing activities of daily living (ADLs) (without limitation, with limitation), and mobility difficulties (without difficulty, with difficulty) were evaluated. Physical activity (active, inactive) was also assessed. The EURO-D scale with 12 items, a specific and validated tool for measuring the presence of depressive symptoms in older adults in European countries, was used with a maximum score of 12 (very depressed) and a minimum of 0 (not depressed), and a cutoff point of 4 indicated the presence of depression. This scale has a Cronbach’s alpha coefficient between 0.62 and 0.78 [29]. Additionally, data on the number of doctor visits in the last year (1 to 5, 6 to 10, more than 11), body mass index (underweight, normal weight, overweight, obesity), daily tobacco consumption (yes or no), and alcohol consumption (does not drink or consumes alcohol less than 1–2 times a month, between 1 and 4 days a week, almost every day) were collected.

#### Statistical analysis

Appropriate descriptive measures were used for the characteristics of the CASP-12, SDHs, and participants’ sociodemographic and clinical variables. Central tendency and dispersion measures were employed for numerical variables, while absolute frequencies and relative percentages were used for categorical variables. Bivariate analysis was conducted between the dependent variable CASP-12 and the independent variables of DSS, considering European regions and sex in each of the three waves, as well as differences between pairs of regions.

To examine the effect of SDHs on population QoL from a sex perspective, the variables used in the models were selected based on author consensus and a literature review. The linearity of the selected variables was evaluated, and a skewed distribution was observed in the number of children and grandchildren. These variables were grouped into categories following suggestions from spline smoothing regression. Furthermore, multicollinearity among predictor variables was evaluated using the generalized variance inflation factor (gVIF), and a maximum gVIF of 1.22 was found, indicating no multicollinearity issues.

Multivariate models were performed using linear mixed-effects models with the lmer function from the lme4 package in R software. An initial model was constructed that included CASP-12 values as the outcome and all explanatory variables. To account for longitudinal measures (three consecutive interviews), participant identification was included as a random effect. Then, one by one, variables that did not show statistical significance were removed (backward method), and the change in the coefficients of the remaining variables was evaluated. None of the remaining coefficients in the final model experienced a change greater than 10% compared to the full model. Although the number of grandchildren was not statistically significant, the variable was retained in the models due to its effect on age and consecutive visits. Similarly, sex was also not statistically significant, but it was retained in the final models to examine its interaction effect with other variables. Data analysis was performed using SPSS 25 and R version 4.1.0 software. A significant difference was considered when the p value was less than 0.05.

### Ethical aspects

The Ethics Committee of the Max Planck Society for the Advancement of Science has thoroughly reviewed the materials of the SHARE project, including wave 5 and its follow-up waves (waves 6 and 7).

## Results

### SDHs and sociodemographic and clinical variables stratified by sex

In the fifth wave differences were observed between men and women when analyzing the SDHs. The average age for women was 63.7 (SD 10.2) years, while for men, it was 64.8 (SD 9.4) years. The most represented age group was the 50–64 age group, encompassing 54.4% of women and 50.0% of men. Regarding education, 37.0% of women had a low education level compared to 32.9% of men (p<0.001). Economic difficulty was observed in 30.5% of women and 26.7% of men (p<0.001). Additionally, over 90% of respondents of both sexes were native to the country where the interview took place. The CASP-12 score was higher among men than among women (p<0.001). For complete data, refer to Table 1.

When analyzing the sociodemographic and clinical variables, notable differences were found between men and women. Regarding marital status, the majority of participants of both sexes were married, but there was a higher proportion of widows (13.4%) than widowers (4.5%) (p<0.001), as well as a higher proportion of women living alone than men (19.4% vs. 12.6%) (p<0.001). Self-perceived health was significantly lower among women. Although limitations in activities of daily living (ADLs) were infrequent and participants of both sexes had few mobility difficulties, they were more common among women (p<0.001). Regarding depression (EURO-D), a significant difference was observed between women (30.3%) and men (17.5%). Physical inactivity was more frequent among women (49.8%) than among men (42.4%) (p<0.001). Additionally, tobacco and alcohol consumption were significantly higher among men than among women. For complete data, refer to Table 2.

### SDHs and QoL differentiated by sex and European region in the 5th wave of recruitment

When analyzing the SDHs and QoL, significant differences were observed in terms of sex and European region. Regarding SDHs, the southern region had an older population of people of both sexes, with 6% of women over 85 years of age. In contrast, the continental and northern regions had the lowest proportions of this age group, at 2.3% and 2.4%, respectively (p<0.001). In terms of educational level, the southern region showed the highest percentage of women with a low education level, 76.3% of the respondents, while in the northern region, this proportion was 23.7% (p<0.001). In relation to economic difficulty in making ends meet, the eastern and southern regions had the highest percentages, especially among women, with 50.8% and 46.1%, respectively, compared to 13.4% in the northern region and 23.7% in the continental region (p<0.001). In terms of nativity, the continental region had a proportion of 16% of respondents of both sexes who were not native to the country of residence, while in the southern region, this percentage was 4.8% (p<0.001).

Regarding QoL measured by the CASP-12, 49.0% and 40.7% of women in the southern and eastern regions, respectively, reported having low QoL. This contrasts with 14.4% in the northern region and 25% in the continental region (p<0.001). This trend was also observed among males. For complete data, refer to Table 3.

### Relationship between QoL and SDHs differentiated by sex, region, and consecutive waves

In the fifth wave, a lower QoL score was observed in the southern region of Europe for both sexes, with the most significant differences observed between women in the southern region and women in the northern region, 34.9 (SD 6.5) vs. 40.7 (SD 5.2) (p<0.001), respectively. This trend also persisted in subsequent waves (6 and 7). In all European regions, men obtained higher QoL scores than women, although the sex difference was smaller in the northern region of Europe in all waves (p<0.005). Furthermore, the score decreased with age for both sexes and in all European regions and consecutive waves (p<0.001).

Regarding educational level, women in the southern region of Europe with a low level of education obtained the lowest QoL score, 34.3 (SD 6.6), compared to men, 35.6 (SD 6.3) (p<0.005), and this trend persisted during the follow-up. The variable that showed the greatest significant sex differences in QoL scores in all European regions and consecutive waves was economic difficulty, which was notably lower in the southern and eastern regions and among women (p<0.001). Additionally, in all waves, both native men and women obtained a better score than immigrants, except in the southern region of Europe. For complete data, refer to Table 4.

### Relationship between score QoL and covariates differentiated by sex and European region.

In the fifth wave, both male and female respondents from all European regions who lived with a partner obtained a higher QoL score than those who lived alone. The score was always higher for men living with a partner, except in the northern region. The difference was significantly greater between women and men in the southern region.

Self-perceived health was the variable with the greatest differences in QoL scores; women in the southern region scored lower in excellent or very good quality of life compared to women in the northern region, 38.5 (SD 5.4) vs. 42.6 (SD 3.6) (p<0.05), respectively. Women with worse objective health data obtained lower QoL scores than men in all regions, with these sex score differences being greater in the southern and eastern regions of Europe (p<0.05). The presence of depression showed a significant QoL score decrease for both women and men in all regions, with it being more notable among women and in the southern region (p<0.05).

Regarding lifestyle, no significant differences in QoL scores related to smoking habits were found between regions or sexes, except in the continental region and Northern Europe, where nonsmoking men obtained a better QoL score. Regarding alcohol consumption, individuals of both sexes who consumed alcohol moderately (1–4 days/week) obtained the highest scores in all regions. Women who consumed alcohol daily in the eastern region of Europe obtained a higher score, 39.6 (SD 5.7), compared to men, 36.8 (SD 6.5) (p<0.05). For complete data, refer to Table 5.

### Multivariate analysis of QoL evolution in relation to SDHs, European region, and consecutive waves

The multivariate analysis examined the relationship between the CASP-12 scores across all waves as the dependent variable and the SDH, European region, and consecutive wave variables. The analysis was adjusted for significant sociodemographic and clinical covariates.

Women experienced a significant decrease in QoL score from wave 5 to wave 7 compared to men (β: −0.196, 95% CI: −0.345; −0.047 vs. β: 0.038, 95% CI: −0.122; 0.197). The interaction between sex and consecutive waves achieved a significance of 0.019, indicating that the decrease in QoL score was more pronounced among women than among men over time.

Regarding age, both women and men in older age groups reported worse QoL than those in younger age groups. However, the difference was more pronounced among women than among men (β: −1.611, 95% CI: −2.083; −1.140 vs. β: −1.051, 95% CI: −1.558; −0.545).

During follow-up, participants of both sexes without economic difficulties obtained a higher QoL score than those who reported difficulties. This difference was greater among women than among men (β: 2.417, 95% CI: 2.234; 2.601 vs. β: 2.038, 95% CI: 1.833; 2.243), with a p for interaction of 0.0057.

Participants of both sexes with a high level of education achieved a better QoL score than those with a low level of education during follow-up. This difference was more significant among men than among women (β: 0.455, 95% CI: 0.193; 0.717 vs. β: 0.252, 95% CI: 0.003; 0.501), indicating that educational level had a greater influence on QoL among men than among women over time.

Differences in QoL scores were observed among interviewees of both sexes according to their region of residence. These differences were more pronounced among women and between the southern and northern regions (β: 2.709, 95% CI: 2.403; 3.015) than among men (β: 2.224, 95% CI: 1.896; 2.551), with a p for interaction of 0.009. Living in a rural area was a protective factor in QoL evolution for women compared to men. No significant association was found between place of residence and QoL among men. For complete data, refer to Table 6.

## Discussion

This study investigated SDHs and QoL among people aged 50 years and older in 13 European countries from a sex perspective. This study was conducted before the COVID-19 pandemic. The results revealed significant differences in the evolution of QoL between men and women during follow-up.

Consistent with the findings of previous research using the SHARE cohort [30], our findings support evidence that women experienced a significant decline in their QoL compared to men over time, more significantly in the Southern European region.

Our findings suggest that not facing economic difficulties has a protective effect on the evolution of QoL. This statement is supported by a study by Niedzwiedz et al. [31] in 2014, which demonstrated a significant association between lifelong socioeconomic position and life satisfaction in early old age and revealed differences in this association between countries with different welfare systems. In our study, economic hardships were also related to lower QoL scores, especially in Southern and Eastern European countries during the follow-up period, and this effect was more evident among women. This finding is consistent with that of previous research, such as that of Conde-Sala et al. in 2017 [32], suggesting that QoL is related to social welfare regimes. These regimes are more limited in Eastern and Southern European countries compared to Nordic and continental countries and have a protective effect on the evolution of QoL, especially among women [33]. We also found that being native to the country where the interview took place was a protective factor in the evolution of quality of life for women, in line with previous studies that found that migrant women experience triple discrimination based on ethnicity, sex, and class [34] that have significant implications for their QoL [35], which is not the case for men.

In this study, we observed that lower levels of education were correlated with worse QoL, especially in Southern European countries and among women. These results are consistent with those of other studies, such as that by Rivas et al. [36] in 2011, which found an association between lower education levels and worse perceived QoL. The southern region had a higher prevalence of depression among older women, which was directly related to lower QoL, in line with the findings of a meta-analysis conducted by Zhao et al. in 2012 [37].

No significant differences were found regarding place of residence, except among women, for whom living in a rural area had a protective effect on the evolution of QoL compared to living in a large city. These results differ from those of previous research, such as that of Lenehan et al. [38] in 2020, in which their meta-analysis showed that older adults living in rural areas had significantly lower health-related QoL than those living in urban areas.

Regarding lifestyle, differences were observed in tobacco consumption between men and women in all European regions and waves, with a higher prevalence among men. These findings have been shown in previous studies conducted with the SHARE cohort [39] and data from the European Health Survey [40]. In our study, we found that moderate alcohol consumption was associated with higher QoL scores, especially in the Southern and Eastern European regions. This could be explained by factors such as geographic location, alcohol availability, and social norms that normalize alcohol consumption, especially among men [40]. Physical activity was lower among women during the follow-up period and was directly related to a decrease in QoL scores, in line with a recent study that provided strong evidence that regular physical activity has a positive impact on the QoL of older adults [41].

Among the strengths of this study, it is worth noting that a representative sample of 13 European countries was used, and the respondents participated in three consecutive waves, allowing for an understanding of their individual aging trajectories with a sex perspective. Additionally, the use of a multidisciplinary database provided a broad and cross-sectional view of the respondents. Finally, it is important to mention that grouping by European regions allowed for the identification of differences between them, as well as the association between SDHs and sex-based differences.

However, this study also has some limitations that should be considered when interpreting the results. First, there is a possibility that the collected data may be biased due to the use of self-report measures. Additionally, case loss between each wave and the selection of only those respondents who participated in all three consecutive waves may limit the external validity of this study. It is also important to note that the data were collected before the COVID-19 pandemic, so the results may differ considerably from those obtained in studies conducted after the pandemic.

It is necessary to continue investigating health inequities. To achieve this goal, it is crucial to implement social welfare policies that include increased investment in education, social benefits to reduce poverty, and government support for older adults in general and for women, especially in countries with less beneficial welfare systems. This economic investment in support would result in lower future investment in the health care system and contribute to reducing the significant differences observed between European countries and regions, as well as between sexes.

## Conclusions

During the follow-up period, women had lower QoL scores than men. This disparity was particularly notable in the eastern and southern regions of Europe, where the social welfare model is less effective, compared to the northern region, where the sex gap was smaller.

The main SDHs associated with lower QoL scores, considering the sex perspective during the follow-up period, were advanced age, economic hardship (more evident in the southern and eastern regions of Europe), educational level, and geographic location within Europe.

Furthermore, a higher prevalence of depression was found among women and in the southern region of Europe, which was also associated with a decrease in QoL scores. Physical activity in the female population was lower throughout the follow-up period, which was directly related to a decrease in QoL scores.

This analysis allowed the identification of sex inequalities in the QoL of individuals over 50 years of age in Europe, as well as the determinants that influence them.

It is expected that the results of this study will contribute to informing policies and practices that promote sex equality and improve the quality of life of older adults in Europe.

## Figures and Tables

**Table 1.** Social determinants of health, differentiated by sex in wave 5 of recruitment and changes in CASP-12.

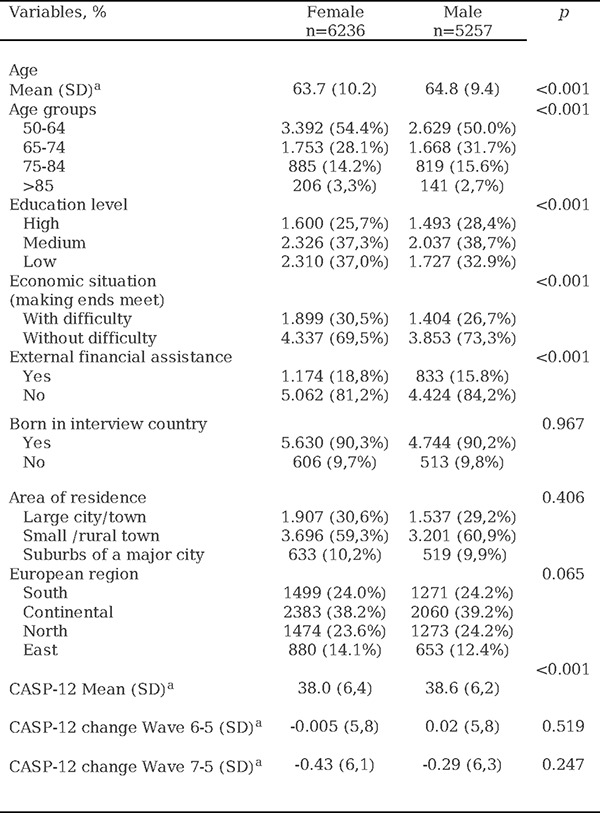

Notes:

a Mean (standard deviation)

CASP-12: Quality of Life and Well-being Index

**Table 2.** Sociodemographic and clinical variables, differentiated by sex in wave 5 of recruitment

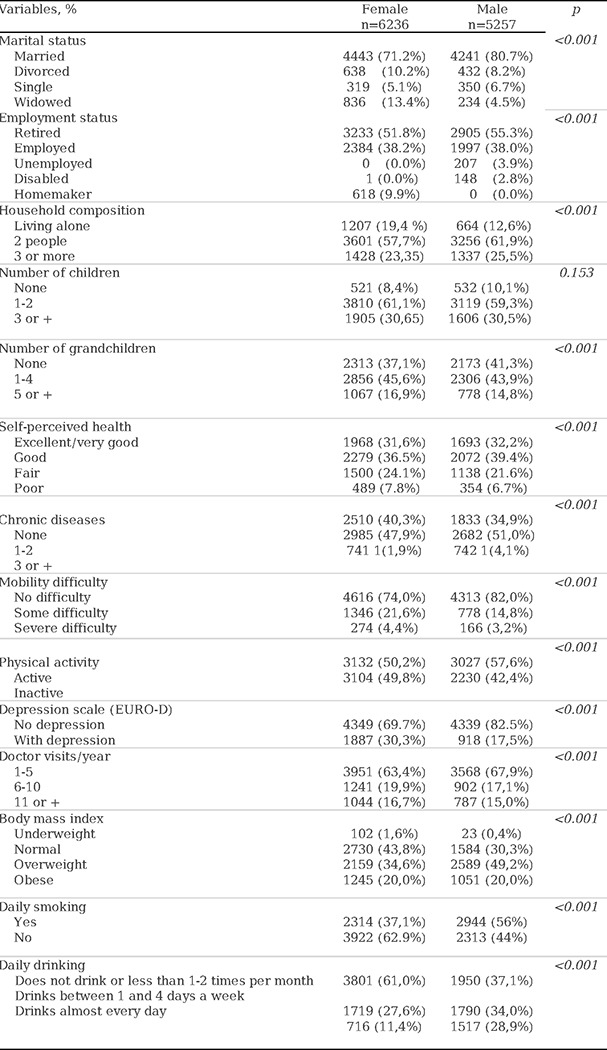

**Table 3.** Social determinants of health and quality of life differentiated by sex and European region in wave 5 of recruitment.

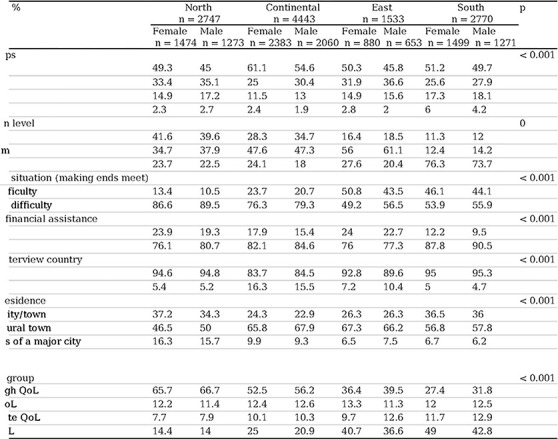

Notes:

CASP-12: Quality of life and well-being index.

**Table 4.** Relationship between score QoL and SDHs differentiated by sex, region, and consecutive waves

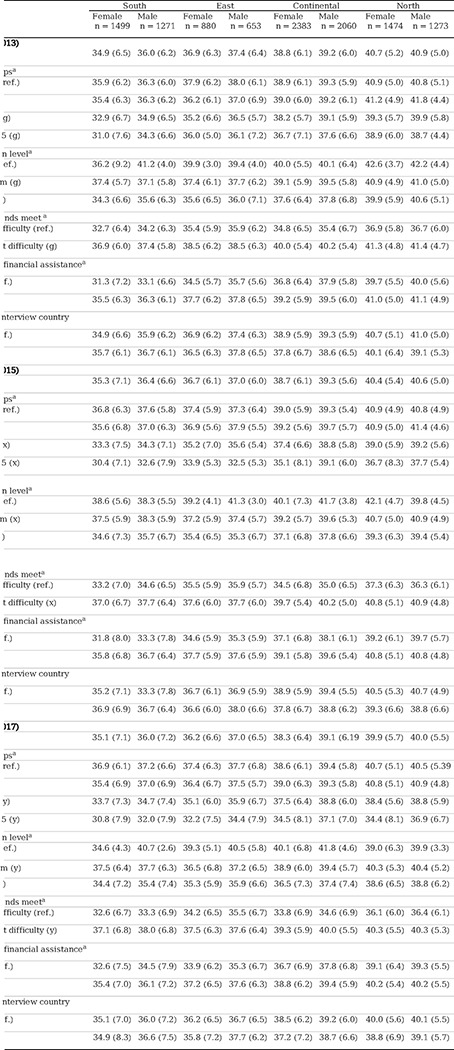

Abbreviations: Ref.: reference.

a Mean, (standard deviation).

Notes:

European regions: South: Spain, Italy; East: Slovenia, Estonia, Czech Republic; Continental: Austria, Germany, Belgium, France, Luxembourg, Switzerland; North: Denmark, Sweden.

(f) In each of the three waves, differences between pairs of regions (South vs. East; South vs. Continental; South vs. North; East vs. Continental; East vs. North; Continental vs. North) p value < 0.001.

Selected category versus reference category p value < 0.05, (g) in Wave 5, (x) in Wave 6, and (y) in Wave 7.

**Table 5.** Relationship between score QoL and covariates differentiated by sex and European regions in Wave 5 (2013)

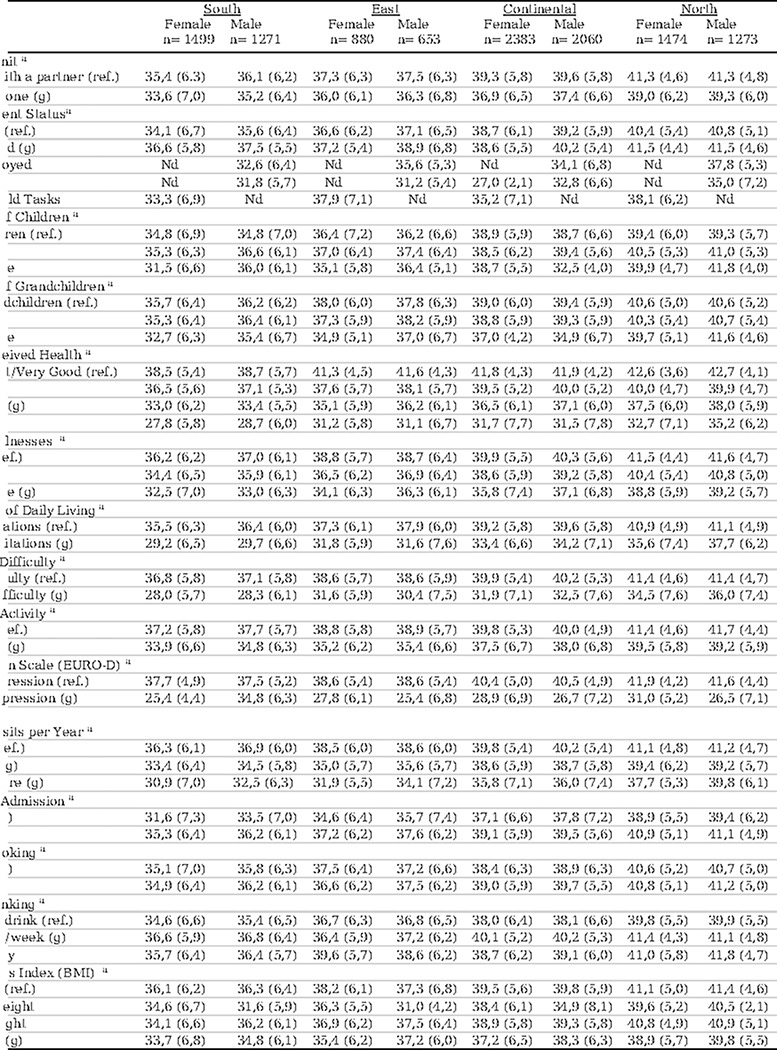

ions: Ref.: reference.

standard deviation).

category versus reference category p value < 0.05 (g)

**Table 6.** Multivariate analysis of social determinants of health by sex, European region, and consecutive waves.

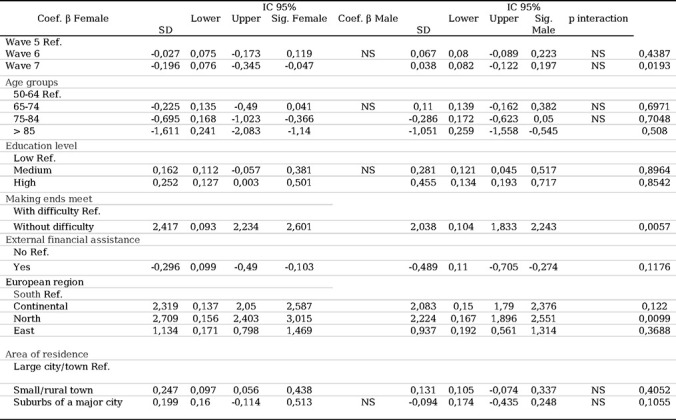

Abbreviations: Ref.: reference.

## Data Availability

This research utilizes data from waves SHARE 5, 6, and 7 (DOIs: 10.6103/SHARE.w5.700, 10.6103/SHARE.w6.700, 10.6103/SHARE.w7.700). www.share-project.org
